# A Multimethod Assessment of a New Customized Heat-Treated Nickel–Titanium Rotary File System

**DOI:** 10.3390/ma15155288

**Published:** 2022-07-31

**Authors:** Emmanuel J. N. L. Silva, Jorge N. R. Martins, Natasha C. Ajuz, Henrique S. Antunes, Victor T. L. Vieira, Francisco M. Braz Fernandes, Felipe G. Belladonna, Marco A. Versiani

**Affiliations:** 1 Department of Endodontics, School of Dentistry, Grande Rio University (UNIGRANRIO), Rio de Janeiro 21210-623, Brazil; natajuz@hotmail.com (N.C.A.); antunesendo@gmail.com (H.S.A.); victortalarico@gmail.com (V.T.L.V.); 2 Department of Endodontics, Fluminense Federal University, Rio de Janeiro 24220-900, Brazil; felipebelladonna@hotmail.com; 3 Department of Endodontics, Rio de Janeiro University (UERJ), Rio de Janeiro 20550-013, Brazil; 4 Faculdade de Medicina Dentária, Universidade de Lisboa, 1600-277 Lisboa, Portugal; jnr_martins@yahoo.com.br; 5 Unidade de Investigação em Ciências Orais e Biomédicas (UICOB), Faculdade de Medicina Dentária, Universidade de Lisboa, 1600-277 Lisboa, Portugal; 6 Centro de Estudo de Medicina Dentária Baseada na Evidência (CEMDBE), Faculdade de Medicina Dentária, Universidade de Lisboa, 1600-277 Lisboa, Portugal; 7 CENIMAT/I3N, Department of Materials Science, NOVA School of Science and Technology, Universidade NOVA de Lisboa, 2829-516 Caparica, Portugal; fbf@fct.unl.pt; 8 Dental Specialty Center, Brazilian Military Police, Belo Horizonte 30350-190, Brazil; marcoversiani@yahoo.com

**Keywords:** differential scanning calorimetry, endodontics, energy-dispersive X-ray spectroscopy, micro-computed tomography, root canal therapy, scanning electron microscopy

## Abstract

This study aimed to compare three endodontic rotary systems. The new Genius Proflex (25/0.04), Vortex Blue (25/0.04), and TruNatomy (26/0.04v) instruments (n = 41 per group) were analyzed regarding design, metallurgy, and mechanical performance, while shaping ability (untouched canal walls, volume of removed dentin and hard tissue debris) was tested in 36 anatomically matched root canals of mandibular molars. The results were compared using one-way ANOVA, post hoc Tukey, and Kruskal–Wallis tests, with a significance level set at 5%. All instruments showed symmetrical cross-sections, with asymmetrical blades, no radial lands, no major defects, and almost equiatomic nickel–titanium ratios. Differences were noted in the number of blades, helical angles, cross-sectional design, and tip geometry. The Genius Proflex and the TruNatomy instruments had the highest and lowest R-phase start and finish temperatures, as well as the highest and lowest time and cycles to fracture (*p* < 0.05), respectively. The TruNatomy had the highest flexibility (*p* < 0.05), while no differences were observed between the Genius Proflex and the Vortex Blue (*p* > 0.05). No differences among tested systems were observed regarding the maximum torque, angle of rotation prior to fracture, and shaping ability (*p* > 0.05). The instruments showed similarities and differences in their design, metallurgy, and mechanical properties. However, their shaping ability was similar, without any clinically significant errors. Understanding these characteristics may help clinicians to make decisions regarding which instrument to choose for a particular clinical situation.

## 1. Introduction

The technology behind the metallurgy of nickel–titanium (NiTi) alloys allowed for the development of new rotary endodontic files with a variety of designs and improved efficiency and safety [1], aiming to reduce iatrogenic mishaps, such as deviation or perforation [2]. Currently, shaping procedures using NiTi rotary instruments are more predictable and easier when compared to manual preparation with stainless-steel files [1,2]. The NiTi alloys used to produce endodontic instruments have an almost equiatomic ratio of nickel and titanium elements [3,4] and may have three microstructural phases, namely austenite, R-phase, and martensite, responsible for their mechanical behavior [3,5]. The conventional superelastic NiTi alloy has a predominant austenite structure at both room (20 °C) and body (37 °C) temperatures, and for this reason, it is relatively stiff, hard, and has limited flexibility. To overcome this limitation, new manufacturing processes using heat treatment have been developed to produce endodontic NiTi instruments with larger amounts of the stable martensite phase [5]. In its martensite form, the NiTi alloy is soft, ductile, and can be easily deformed [3,5], while the R-phase transformation commonly appears as an intermediate phase in most of the commercially available NiTi wires [6]. Compared to austenitic instruments, it has been reported that heat-treated NiTi instruments have increased cyclic fatigue resistance, strength [7,8,9], and flexibility, presenting lower bending loads in the bending tests [8,9,10].

In the last decade, the optimized properties of heat-treated NiTi instruments led companies to launch several new rotary systems on the market. Vortex Blue (Dentsply Sirona, Baillagues, Switzerland) was introduced in 2011, and the proprietary heat treatment improved its mechanical properties compared to its predecessor, manufactured with M-Wire alloy [7]. The heat-treated TruNatomy rotary instruments (Denstply Sirona, Ballaigues, Switzerland) have a variable taper with an off-centered parallelogram cross-sectional design, and studies have reported its ability to preserve the radicular dentin during root canal mechanical preparation [11,12]. Genius Proflex (Medidenta, Las Vegas, NV, USA) is a recently launched multi-file rotary system composed of instruments with different cross-sections and submitted to distinct heat treatments, resulting in active blades with different colors (purplish, blueish, and yellowish), aiming to ensure a balance between flexibility and resistance, depending on the metal mass of each instrument in the series (https://bit.ly/3rgSqEH (accessed on 25 May 2022)). Thus far, there is no available scientific evidence to support its efficiency or safety. Therefore, the aim of this study was, by using a multimethod approach, to evaluate the design, metallurgy, mechanical performance, and shaping ability of the Vortex Blue, TruNatomy, and Genius Proflex rotary instruments. The null hypothesis to be tested in the present research was that there would be no differences among these instruments regarding the evaluated properties.

## 2. Materials and Methods

**Figure 1:**
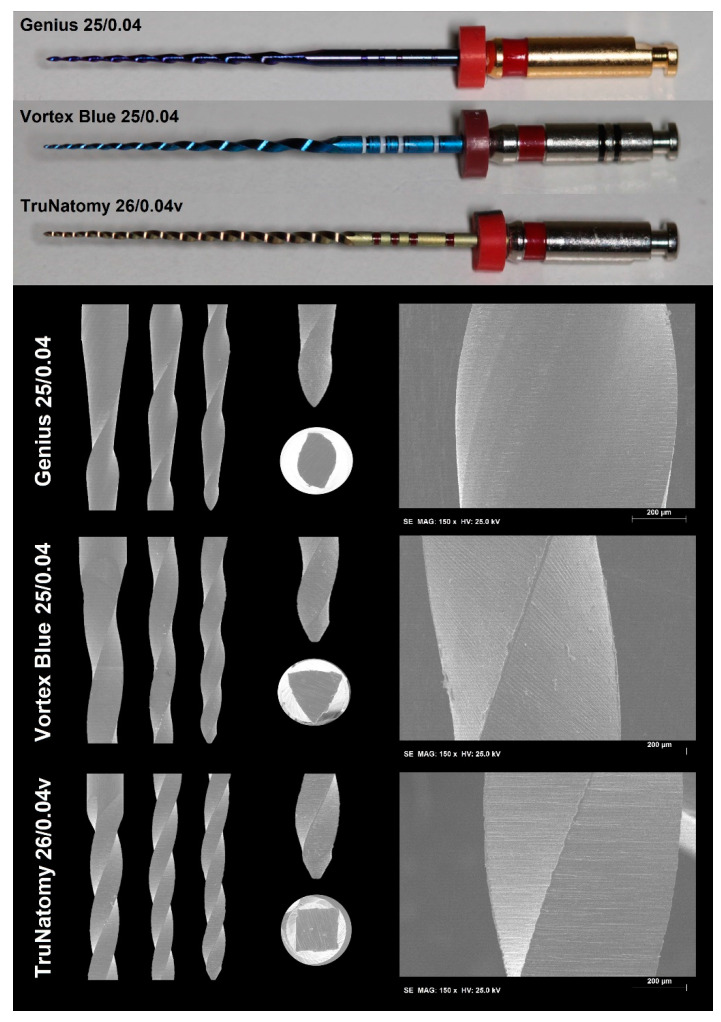
Tested instruments and their design and surface finishing. Macroscopic analyses of the tested instruments (top) showed a higher number of blades in the TruNatomy and distinct colors of the alloy among them. SEM evaluation (bottom) revealed that all instruments have asymmetrical blades, no radial lands and different symmetrical cross sections (square: TruNatomy; triangular: Vortex Blue; S-shaped: Genius Proflex). The tips were non-active, with distinct geometry and transition angles. All surfaces had parallel manufacturing marks, with few irregularities.

New 25-mm NiTi instruments (n = 123) from 3 rotary systems (41 per group; Genius Proflex (25/0.04), TruNatomy (26/0.04v), and Vortex Blue (25/0.04)) (Figure 1) were compared in relation to design, metallurgical characteristics, and mechanical behavior. In addition, 48 instruments (16 per group) were employed for testing the shaping ability of each system in root canals of extracted mandibular molars. Instruments were previously examined under a stereomicroscope (×13.6 magnification; Opmi Pico, Carl Zeiss Surgical, Oberkochen, Germany) looking for defects that would exclude them from being tested, but none were excluded. 

### 2.1. Instrument Design 

The number of active blades (in units) and the helical angles (in degrees) at the 6 most coronal flutes of 6 randomly selected endodontic files from each system were assessed under stereomicroscopy (×13.6 magnification; Opmi Pico) using the ImageJ v1.50e software (Laboratory for Optical and Computational Instrumentation, Madison, WI, USA). These same instruments were further imaged in a conventional scanning electron microscope (Hitachi S-2400, Hitachi, Tokyo, Japan) at different magnifications (×100 and ×500) to evaluate their blade design (radial lands and symmetry), cross-sectional shape, tip geometry (active or non-active), and surface finishing.

### 2.2. Metallurgical Characterization 

The semi-quantitative elemental analysis of 3 instruments from each tested system was carried out to evaluate the nickel and titanium ratio, or the presence of any other element, using a scanning electron microscope (S-2400; Hitachi) mounted with an energy-dispersive X-ray spectroscopy (EDS) device (Bruker Quantax; Bruker Corporation, Billerica, MA, USA) set at 20 kV and 3.1 A. The analysis was performed for each instrument at a 25-mm distance from a surface area of 400 µm^2^ using a proper software with ZAF correction (Systat Software Inc., San Jose, CA, USA). 

The differential scanning calorimetry (DSC) method (DSC 204 F1 Phoenix; Netzsch-Gerätebau GmbH, Selb, Germany) was used to determine the phase transformation temperatures of the NiTi alloy following the guidelines of the American Society for Testing and Materials [13]. Fragments of 2 to 3 mm in length (5–10 mg), removed from the coronal active blade of 2 instruments from each system, were exposed for 2 min to a chemical etching consisting of a mixture of 45% nitric acid, 25% hydrofluoric acid, and 30% distilled water. Then, they were mounted in an aluminum pan inside the DSC device, with an empty pan serving as control. The thermal cycle was performed under gaseous nitrogen atmosphere at a pace of 10 °C/min with temperatures ranging from −150 °C to 150 °C. Phase transformation temperatures were analyzed by the Netzsch Proteus Thermal Analysis software (Netzsch-Gerätebau GmbH). For each group, the DSC test was performed twice to confirm the results. Tested instruments included TruNatomy size 26/0.04v, Vortex Blue size 25/0.04, and the whole set of Genius Proflex instruments (sizes 25/0.06, 13/0.03, 17/0.05, 25/0.04, and 35/0.04) due to differences in their heat treatment, as claimed by the manufacturer (https://bit.ly/38DxX6J (accessed on 25 May 2022)). 

### 2.3. Mechanical Tests 

The mechanical performance of the selected systems was evaluated through cyclic fatigue, torsional resistance, and bending tests. For each test, the sample size was calculated with an alpha-type error of 0.05 and a power of 80%, based on the highest difference between 2 systems after 6 initial measurements. For the time to fracture (TruNatomy vs. Genius Proflex; effect size of 217.8 ± 118.8), maximum torque (TruNatomy vs. Vortex Blue; effect size of 0.15 ± 0.22), angle of rotation (TruNatomy vs. Genius Proflex; effect size of 6.2 ± 48.2), and maximum bending load (TruNatomy vs. Vortex Blue; effect size of 67.7 ± 37.2), the final sample sizes of 6, 36, 949, and 6 instruments were determined, respectively. Even though 36 and 949 instruments were calculated for the maximum torque and angle of rotation, a final sample size of 10 instruments per group was defined for each parameter, since a difference only identifiable in that large a sample size can be considered of little clinic relevance.

The cyclic fatigue test was conducted on a non-tapered stainless steel curved tube apparatus (radius of 6 mm and 86° degree angle) using glycerin as a lubricant, according to previous studies [8,9,14]. The tested instruments were adapted to a 6:1 reduction handpiece (Sirona Dental Systems GmbH, Bensheim, Germany) and activated at static mode by a torque-controlled motor (VDW Silver; VDW GmbH) set at 400 rpm and 2.0 N (Genius Proflex), 500 rpm and 1.5 N (TruNatomy), and 500 rpm and 1.0 N (Vortex Blue), according to the manufacturers’ directions. The test was conducted at room temperature (20 °C) following the guidelines of the American Society for Testing and Materials regarding tension testing of superelastic NiTi materials [15]. Fracture was detected by both auditory and visual inspection. The time to fracture was recorded in seconds using a digital chronometer, and the fragment size was measured in millimeters with a digital caliper for experimental control. Torsional and bending resistance tests were performed according to international standards [16,17]. In the torsional test, instruments were clamped 3 mm from their tip and rotated clockwise at a constant pace of 2 rotations per minute to assess the maximum torque (measured in N.cm) and the angle of rotation (recorded in degrees) prior to fracture. In the bending test, each instrument was mounted in the file holder of the motor and positioned at 45° in relation to the floor, while it was attached to a wire (3 mm from its tip) connected to a universal testing machine (Instron 3400; Instron Corporation, Canton, MA, USA). The maximum load needed for a 45° displacement of the instrument, using a load of 20 N and 15 mm/min of constant speed, was recorded in gram-force (gf). 

### 2.4. Shaping Ability 

After approval of this research project by the local ethics committee (Protocol CE-FMDUL 13/10/20), 120 two-rooted mandibular molars with fully formed apices were randomly selected from a pool of extracted teeth and initially scanned at a pixel size of 11.93 μm in a micro-computed tomographic device (micro-CT) (SkyScan 1173; Bruker-microCT, Kontich, Belgium) set at 70 kV, 114 µA, rotation of 360° with steps of 0.7°, using a 1 mm thick aluminum filter. The first step in the image acquisition involved fixing the specimen on a sample holder with dental wax to avoid movement during scanning. The acquired projections were reconstructed into axial cross-sections using standardized parameters of smoothing (1), attenuation coefficient (0.05–0.007), beam hardening (20%), and ring artifact (5) corrections (NRecon v.1.6.9; Bruker-microCT). A three-dimensional (3D) model of the internal anatomy of each tooth was created (CTAn v.1.14.4; Bruker-microCT) and qualitatively evaluated (CTVol v.2.2.1; Bruker-microCT) regarding root canal configuration. Then, and considering teeth with the same working length from cementoenamel junction to the apex, and the same volume and surface area from the mesial and distal canals, were calculated, within these two anatomic landmarks. Based on these parameters, specimens were anatomically matched to create 3 groups of 4 teeth (12 canals per group) that were randomly assigned to an experimental group according to the preparation system: Genius Proflex, TruNatomy, and Vortex Blue.

After access cavity preparation, apical patency was confirmed with a size 10 K-file (Dentsply Sirona Endodontics) and the glide path was performed using a size 15 K-file (Dentsply Sirona Endodontics) up to the working length (WL), established 1 mm from the apical foramen. In the Genius Proflex group, coronal flaring was performed with a size 25/0.06 instrument (350 rpm, 2.5 N.cm), followed by instruments in sizes 13/0.03 (250 rpm, 1.5 N.cm) and 25/0.04 (400 rpm, 2 N.cm) up to the WL. In the TruNatomy group, all instruments were used at 500 rpm and 1.5 N.cm. After coronal flaring with a size 20/0.08 instrument, instruments of 17/0.02v (Glider) and 26/0.04v (Prime) were used up to the WL. In the Vortex Blue group, instruments of sizes 15/0.04 (500 rpm, 0.7 N.cm), 20/0.04 (500 rpm, 0.7 N.cm), and 25/0.04 (500 rpm, 1 N.cm), were sequentially used up to the WL. Then, in all groups, the distal canals were further enlarged with instruments in sizes 35/0.05 (Genius Proflex group; 400 rpm, 2.5 N.cm), 36/0.03v (TruNatomy group), 30/0.04 and 35/0.04 (Vortex Blue group; 500 rpm, 1.0 N.cm, and 1.3 N.cm, respectively). Instruments were activated by an electric motor (VDW Silver; VDW, Munich, Germany) and used in a slow in-and-out pecking motion of about 3 mm amplitude with light pressure in the apical direction. After 3 pecking motions, the instrument was removed from the canal and cleaned. The WL was reached after 3 waves of instrumentation. Each instrument was used in one tooth and then discarded. Irrigation was performed with a total of 15 mL of 2.5% NaOCl per canal, followed by a final rinse with 5 mL of 17% EDTA (3 min) and 5 mL of distilled water using a syringe fitted with a 30-G NaviTip needle (Ultradent, South Jordan, UT, USA) positioned 2 mm from the WL. All procedures were performed by an experienced operator under magnification (×12.5; ZEISS OPMI Pico, Jena, Germany).

The canals were slightly dried with paper points and a final scan and reconstruction were performed using the previously mentioned parameters. Datasets before and after preparation were co-registered (3D Slicer 4.3.1 software; http://www.slicer.org (accessed on 25 May 2022)) and the shaping ability was assessed by measuring 3 parameters: the volume of dentin removed after preparation (in mm^3^), the volume of hard tissue debris created by the preparation protocols (in mm^3^), and the percentage of unprepared canal walls [18,19]. An examiner blinded to the shaping protocols performed all analyses by excluding canal interconnections and accessory anatomies.

### 2.5. Statistical Analysis

The Shapiro–Wilk and Lilliefors tests were used to verify the normality of the data. Depending on data distribution, results were summarized as mean (standard deviation) or median (interquartile range) values. One-way ANOVA and post hoc Tukey tests were carried out to compare the angle of rotation, untouched canal walls, volume (root canal, removed dentine, hard tissue debris), and surface area (root canal) of the mesial canals, while the Kruskal–Wallis test, combined with the Dunn test, was used to compare the helical angle, time to fracture, maximum torque to fracture, maximum bending load, and volume of removed dentine and hard tissue debris in the distal canal. The significance level was set at 5% (SPSS v25.0 for Windows; SPSS Inc., Chicago, IL, USA).

## 3. Results

### 3.1. Instrument Design

The instrument stereomicroscopic analysis of both number of blades and helical angles showed that the Vortex Blue (11 blades; 17.8° (17.3–18.9°)) had a significantly lower helical angle degree when compared to the TruNatomy (17 blades; 21.3° (19.5–22.1°)) and the Genius (9 blades; 21.7° (19.8–23.1°)) (*p* < 0.05). SEM analysis (Figure 1) revealed that all instruments had asymmetrical blades, with no radial lands, and symmetrical cross sections, with squared (TruNatomy), convex (Vortex Blue), and S-shaped (Genius Proflex) profiles. None of the tips could be identified as active, and the overall geometry and transition angles of the blade varied among the instruments. While the tips of TruNatomy and Vortex Blue instruments were flat at their ends, the Genius Proflex had a bullet-like shape. Under higher magnification, all instruments showed similar surface finishing, with a pattern of parallel marks created by the grinding manufacturing process. It was also possible to observe some metal rollovers on the blades, but Vortex Blue showed more irregularities than the others (Figure 1). 

### 3.2. Metallurgical Characteristics 

**Figure 2:**
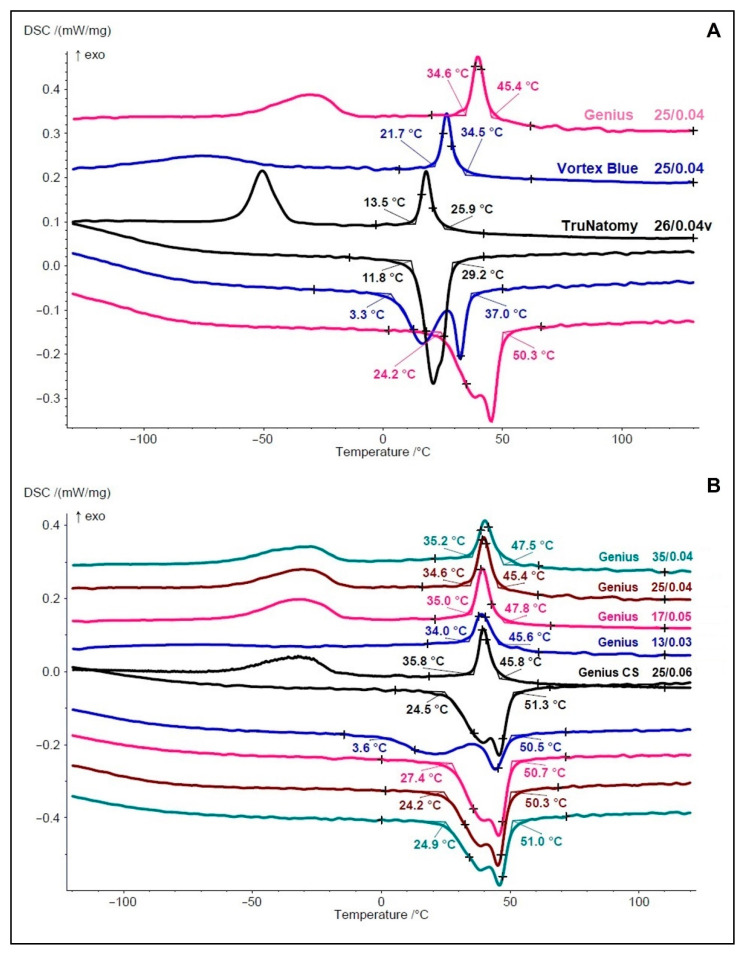
DSC charts showing the phase transformation temperatures at cooling on the top (reads from right to left) and at heating on the bottom (reads from left to right). (**A**) Genius Proflex showed the highest R-phase start (45.4 °C) and finish (34.6 °C) temperatures, while TruNatomy had the lowest (25.9 °C and 13.5 °C, respectively). Genius Proflex also had the highest austenitic start (24.2 °C) and finish (50.3 °C) temperatures. (**B**) Phase transformation temperatures of the Genius Proflex system. Except for the 13/0.03 instrument, which showed a distinct R-phase to martensite B19′ transformation at cooling, all other instruments had similar curves.

EDS/SEM analysis revealed a nearly equiatomic ratio of nickel and titanium elements in the Genius Proflex (1.061), TruNatomy (1.014) and Vortex Blue (1.016) instruments, without any other traceable metal element. DCS analyses (Figure 2A) showed distinct transformation–temperature curves. Although no instrument had full austenitic characteristics at the test temperature (20 °C), Vortex Blue and TruNatomy showed this feature at body temperature (36 °C). The highest (45.4 °C) and lowest (25.9 °C) R-phase start and finish (34.6 °C and 13.5 °C) temperatures were observed in the Genius Proflex and the TruNatomy, respectively (Figure 2A). The Vortex Blue had the lowest austenitic start temperature (3.3 °C) and the Genius Proflex showed the highest austenitic finish temperature (50.3 °C). DSC tests of the Genius Proflex instruments (Figure 2B) demonstrated similar heat treatment among them, with minor differences in R-phase transformation temperatures, in the cooling transformation of martensitic B19′, and in the austenitic transformation during heating curves. Major differences were observed in the heating of the Genius Proflex 13/0.03, with a lower austenitic start (3.6 °C) compared to that of the other instruments (Figure 2B). 

### 3.3. Mechanical Performance 

The Genius Proflex had the highest time (252 s) and cycles (1680) to fracture (*p* < 0.05), while the lowest time (41 s) and cycles (341.7) to fracture were observed with the TruNatomy (*p* < 0.05). The maximum torque and angle of rotation prior to fracture revealed no significant differences among groups (*p* > 0.05). The TruNatomy showed the highest flexibility (108.5 gf) compared to the other tested instruments (*p* < 0.05) (Table 1).

### 3.4. Shaping Ability 

**Figure 3:**
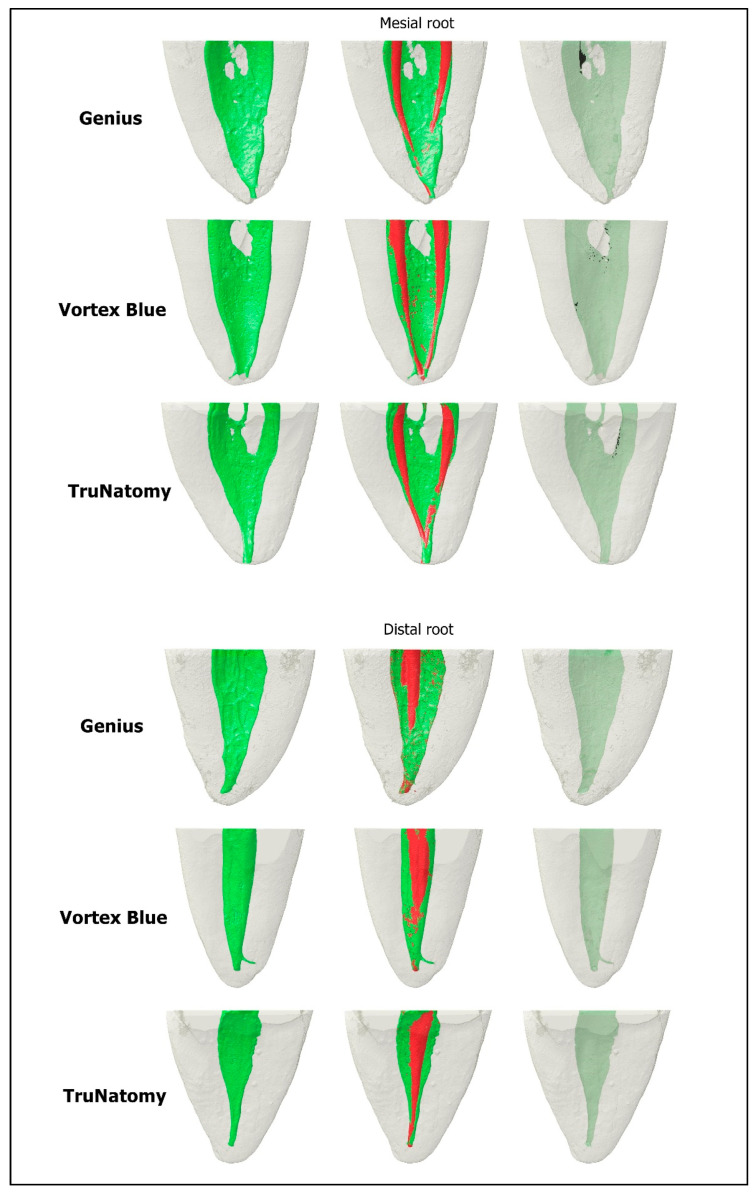
Representative micro-CT 3D models of mesial and distal canals of mandibular molars showing the root canals before (green color) (left column) and after (red color) preparation (central column) using the Genius Proflex, TruNatomy, and Vortex Blue systems. None of the shaping protocols were able to prepare the entire surface area of the root canal walls. Accumulated hard-tissue debris is depicted in black (right column).

The homogeneity of the groups regarding the volume and surface area of the mesial and distal canals was confirmed (*p* > 0.05) (Table 2). No statistically significant differences were observed among the groups in all the tested parameters (*p* > 0.05). Mean percentages of unprepared canal areas ranged from 50.5% to 60.4% in the mesial canal, and from 57.8% to 68.7% in the distal canal (Table 2, Figure 3).

## 4. Discussion

The present investigation, using a multimethod research approach, assessed the overall geometric design, elemental composition, phase transformation temperatures, mechanical behavior, and shaping ability of 3 heat-treated NiTi rotary systems (Genius Proflex, TruNatomy, and Vortex Blue). This methodological approach allows for a more comprehensive assessment regarding the properties of the tested instruments, as it avoids ‘knowledge compartmentalization,’ a phenomenon in which knowledge structures about a specific domain are composed of several separate parts [20]. 

All tests followed strict international guidelines [13,15,16,17] or methodologies with high internal validity [14,18,21,22], enabling a more robust and trustworthy understanding of the systems’ performance. While similarities were observed among the instruments regarding nickel and titanium composition, torsional response (Table 1), and shaping ability (Table 2, Figure 3), differences were observed in the helical angles, number of blades, cross-sections, tip geometry (Figure 1), temperature transition phases (Figure 2), cyclic fatigue, and bending resistance tests (Table 1). Therefore, the null hypothesis was rejected.

Differences in the mechanical behavior of tested instruments should be analyzed considering multiple factors, which may be relevant depending on the test. Since all of the instruments were made from almost equiatomic NiTi alloys, their mechanical behavior may be explained by differences in the design and crystallographic arrangements [3,5], depicted by their distinct phase transformation temperatures (Figure 2A). Considering that all mechanical tests were performed at room temperature (20.0 ± 1 °C), which is inside the instrument’s service temperature range, and in accordance with ASTM recommendations [15], the Rs temperatures of the Genius Proflex (45.4 °C), Vortex Blue (34.5 °C), and TruNatomy (25.9 °C), indicates that none of them had full austenitic characteristics at the test temperature. On the other hand, this baseline temperature tends to increase and approach body temperature (around 36 °C) under clinical conditions. In such cases, the Vortex Blue and TruNatomy instruments may suffer a crystallographic rearrangement leading to a higher increase in the amount of austenitic phase compared to the Genius Proflex. Therefore, the higher martensitic composition and smaller metal core (represented by the S-shaped cross-section and fewer number of blades) of the Genius Proflex instruments, compared to the TruNatomy and the Vortex Blue, could explain its higher cyclic fatigue resistance (Table 1). Unfortunately, the results of the Genius Proflex cannot be compared to the literature, as there is still no scientific publication on its mechanical properties. On the other hand, comparisons between the TruNatomy and the Vortex Blue have shown contrasting results. While in one study [23], no statistical difference was observed in the mean cycles to fracture in the Vortex Blue (523.9) and TruNatomy (436.8), in another study [24], the TruNatomy showed a higher mean number of cycles to fracture (1238.8) compared to the Vortex Blue (529.5). These studies were conducted at body temperature (35–37 °C), and these dissimilarities could be explained by differences in the angles of curvature of the simulated canals (90° vs. 60°). 

Although differences were observed in the cyclic fatigue test, the instruments showed similar results in the torsional resistance assay. This test followed ISO 3630-3631 guidelines [17] that recommend measuring the torsional resistance of an instrument only at 3 mm from its tip. This methodological aspect may explain the observed similarities since, at this specific level, minor differences among the instruments regarding taper (0.04v for TruNatomy, and 0.04 for Vortex Blue and Genius Proflex) are compensated by their dissimilar cross-sectional design and metal core. While little debate exists regarding this methodological aspect, it is possible that analyses of torsional resistance performed at other levels of the instruments may result in different outcomes from those obtained herein.

In this study, an interesting finding was observed in the bending test. While it would be expected that highly flexible instruments would perform better in the cyclic fatigue resistance test, the TruNatomy was the most flexible instrument, but had the lowest cycles to fracture (Table 1). This apparent contradictory result may be explained because of differences in the small diameter of the NiTi wire used to produce the TruNatomy (0.8 mm) compared to the Genius Proflex and Vortex Blue (1.0 mm and 1.2 mm, respectively). Considering that in the bending test, all instruments are fixed in the file holder, the smaller wire can have a direct influence on this result. 

The idea behind the Genius Proflex instruments is to take advantage of different crystallographic phases of the NiTi alloy, depending on the clinical needs. For instance, it would be expected that, during glide path, the instrument suffers a torsional overload, requiring a high torque resistance to avoid unexpected fracture while, for the apical enlargement, especially in curved canals, flexural fatigue resistance would be more relevant than torsional overload. In this way, if all instruments in a set were submitted to the same heat treatment, the accomplished metallurgical changes would be more beneficial to some instruments than to others. Thus, the present study also aimed to analyze all sets of instruments of the Genius Proflex system (25/0.06; 13/0.03; 17/0.05; 25/0.04; 35/0.04) regarding their phase transformation temperatures (Figure 2B). The different transformation temperature profiles in the Genius Proflex customized heat-treated instruments were shown by the glide path instrument (13/0.03), which presented a very distinct R-phase to martensite B19′ transformation at cooling (Figure 2B), compared to the 25/0.06 (yellowish blade color) and 35/0.04 (bluish blade color). 

In addition to the mechanical tests, this study also assessed the shaping ability of the selected rotary systems using the non-destructive micro-CT gold-standard technology. This analytical tool allows for the standardization of specimen selection, avoiding bias related to root canal morphology, and the assessment of several morphometric parameters after root canal preparation [18,19,21]. Although differences were observed in the design and mechanical behavior among the tested instruments (Table 1), all preparation protocols were similar in terms of dentin removed after preparation, hard tissue debris created by the preparation protocols, and unprepared canal walls. Moreover, no instrument fracture or significant deviation from the original canal path could be observed. The similar tip and taper sizes of the tested instruments might explain these results, which are in line with previous studies using instruments with equivalent sizes and tapers [22,25]. In the literature, both the TruNatomy [11,12] and the Vortex Blue [26,27] systems have been evaluated regarding their shaping ability using micro-CT technology. While different methodological strategies were used in these studies, taken together, their outcomes were similar to the present research regarding the large percentage areas of untouched canals walls (TruNatomy: 50%; Vortex Blue: 58.8%) [11,26], the low amount of dentin removal after canal reparateion [12,26], and the small accumulation of hard tissue debris (Vortex Blue system: 0.16 mm^3^) [26]. 

The multimethod research may be seen as one of the main strengths of the present research, which allowed for a more comprehensive assessment of the instruments’ profiles and behaviors. Additionally, the use of DSC allowed a broader understanding of the temperature issue, when compared to tests based on a single temperature, whatever it may be. Among the limitations of the present study are the fact that other relevant tests, such as cutting efficiency, microhardness, and buckling resistance, were not conducted. Future studies using the multimethod approach should include these additional tests to compare and justify this new trend of manufacturers to produce sets of instruments with customized heat-treated NiTi alloys. Knowing the characteristics of these instruments may help the clinicians to take a better decision regarding which instruments to select in a particular clinical situation.

## 5. Conclusions

The Genius Proflex, Vortex Blue, and TruNatomy instruments showed differences regarding the number of blades, helical angles, cross-sectional design, tip geometry, phase transformation temperatures, cyclic fatigue resistance, and flexibility, but were similar in terms of nickel–titanium ratios, maximum torque, angle of rotation prior to fracture, and shaping ability.

## Figures and Tables

**Table 1:** Mechanical behavior of tested instruments shown as mean (standard deviation) and median (interquartile range) values.

System	Cyclic Fatigue		Torsional Test		Bending Test
	Time to Fracture (s)	Cycles to Fracture (NCF)	Maximum Torque (N.cm)	Angle of Rotation (°)	Maximum Load (gf)
TruNatomy 26/0.04v	41.0 (± 8.6) ^a^ 43.5 [31.8–48.0]	341.7 (± 71.7) ^a^ 362.5 [264.6–400.0]	0.76 (± 0.12) ^a^ 0.70 [0.70–0.83]	633.6 (± 40.9) ^a^ 620.5 [602.5–662.3]	108.5 (± 9.5) ^a^ 108.0 [99.5–119.0]
Vortex Blue 25/0.04	80.0 (± 9.1) ^b^ 77.5 [72.3–88.8]	666.7 (± 76.2) ^b^ 645.9 [602.1–739.6]	0.93 (± 0.13) ^a^ 0.90 [0.90–1.00]	589.8 (± 29.0) ^a^ 593.5 [556.0–610.5]	178.8 (± 13.7) ^b^ 180.0 [167.5–186.0]
Genius 25/0.04	252.0 (± 53.7) ^c^ 257.0 [199.8–290.8]	1680.0 (± 357.7) ^c^ 1713.4 [1331.7–1938.3]	0.79 (± 0.27) ^a^ 0.70 [0.58–0.93]	587.3 (± 78.6) ^a^ 609.5 [509.8–659.0]	167.4 (± 16.4) ^b^ 162.0 [158.5–180.5]

Different superscript letters in the same column represent statistically significant differences (*p* < 0.05) among instruments.

**Table 2:** Pre- and post-operative parameters (mean, standard deviation, and range interval) evaluated in mesial (n = 24) and distal (n = 12) root canals of mandibular molars after preparation protocols using 3 rotary systems.

Canal	Parameters		Genius	TruNatomy	Vortex Blue
Mesial	Volume	Before	4.7 ± 1.7 (2.3–6.3)	4.6 ± 1.8 (2.3–6.3)	3.4 ± 1.5 (1.6–5.3)
		After	5.8 ± 1.1 (4.1–6.7)	5.9 ± 1.4 (4.4–7.4)	4.5 ± 1.3 (2.9–6.3)
	Surface area	Before	68.9 ± 11.8 (53.3–81.4)	58.4 ± 16.5 (36.5–71.3)	55.1 ± 17.1 (31.1–70.8)
		After	69.9 ± 12.6 (53.4–83.5)	61.6 ± 12.3 (47.1–73.8)	56.9 ± 16.3 (37.3–74.1)
	Removed dentin	After	1.5 ± 0.6 (0.6–2.3)	1.6 ± 0.4 (1.1–2.2)	1.3 ± 0.07 (1.2–1.4)
	Debris	After	0.037 ± 0.035 (0.003–0.073)	0.013 ± 0.009 (0.004–0.025)	0.014 ± 0.012 (0.002–0.030)
	Unprepared area	After	60.4 ± 17.3 (44.9–77.9)	50.5 ± 24.4 (25.5–75.9)	54.2 ± 24.5 (17.4–69.1)
Distal	Volume	Before	6.1 ± 1.8 (3.9–8.5)	8.2 ± 3.6 (4.7–13.3)	4.6 ± 0.5 (4.1–5.2)
		After	7.4 ± 1.2 (6.3–9.2)	8.8 ± 3.7 (5.9–14.4)	5.7 ± 0.6 (5.1–6.5)
	Surface area	Before	60.5 ± 3.8 (56.8–65.4)	57.3 ± 20.4 (41.4–86.6)	45.3 ± 6.2 (40.9–54.5)
		After	61.9 ± 6.6 (54.9–70.6)	62.4 ± 19.4 (50.9–90.8)	48.2 ± 9.1 (42.1–61.8)
	Removed dentin	After	1.5 ± 1.4 (0.7–3.6)	0.9 ± 0.6 (0.3–1.8)	1.2 ± 0.8 (0.5–2.4)
	Debris	After	0.007 ± 0.010 (0.000–0.021)	0.001 ± 0.003 (0.000–0.005)	0.002 ± 0.003 (0.000–0.007)
	Unprepared area	After	63.6 ± 9.1 (53.4–73.81)	68.7 ± 14.8 (46.8–79.1)	57.8 ± 12.0 (46.6–72.2)

Volume (mm^3^); surface area (mm^2^); removed dentin (mm^3^); debris (mm^3^); unprepared area (%).

## Data Availability

Not applicable.
